# Yellow fever epizootics in non-human primates, Southeast and Northeast Brazil (2017 and 2018)

**DOI:** 10.1186/s13071-020-3966-x

**Published:** 2020-02-19

**Authors:** Maria Angélica Monteiro de Mello Mares-Guia, Marco A. Horta, Alessandro Romano, Cíntia D. S. Rodrigues, Marcos C. L. Mendonça, Carolina C. dos Santos, Maria C. Torres, Eliane S. M. Araujo, Allison Fabri, Everton R. de Souza, Roberta O. R. Ribeiro, Fabiana P. Lucena, Luiz C. A. Junior, Rivaldo V. da Cunha, Rita M. R. Nogueira, Patricia C. Sequeira, Ana M. Bispo de Filippis

**Affiliations:** 1 Laboratório de Flavivírus, Instituto Oswaldo Cruz, FIOCRUZ, Rio de Janeiro, Rio de Janeiro Brazil; 2 Secretaria de Vigilância em Saúde/Ministério da Saúde, Brasília, Brazil; 3 Laboratório Municipal de Saúde Pública (LASP), Instituto Municipal de Medicina Veterinária Jorge Vaitsman, Rio de Janeiro, Brazil; 4 Coordenação de Vigilância em Saúde e Laboratórios de Referência da Fundação Oswaldo Cruz, FIOCRUZ, Rio de Janeiro, Brazil

**Keywords:** Yellow fever, Epizootics, Non-human primates, Molecular diagnosis, Outbreak

## Abstract

**Background:**

Yellow fever (YF) is a severe, infectious, but non-communicable arboviral hemorrhagic disease. In the last decades, yellow fever virus (YFV) infections have been prevalent in endemic areas in Brazil, affecting human and non-human primate (NHP) populations. Monitoring of NHP infection started in 1999, and reports of epizootic diseases are considered important indicators of viral transmission, particularly in relation to the sylvatic cycle. This study presents the monitoring of YFV by real-time RT-PCR and the epidemiological findings related to the deaths of NHPs in the south-eastern states and in the north-eastern state of Bahia, during the outbreak of YF in Brazil during 2017 and 2018.

**Methods:**

A total of 4198 samples from 2099 NHPs from south-eastern and north-eastern Brazilian states were analyzed by real-time reverse transcription polymerase chain reaction (rtRT-PCR).

**Results:**

A total of 4198 samples from 2099 NHPs from south-eastern and north-eastern Brazilian states were collected between 2017 and 2018. The samples were subjected to molecular diagnostics for YFV detection using real-time reverse transcription polymerase chain reaction (rtRT-PCR) techniques. Epizootics were coincident with human YF cases. Furthermore, our results showed that the YF frequency was higher among marmosets (*Callithrix* sp.) than in previous reports. Viremia in species of the genus *Alouatta* and *Callithrix* differed greatly.

**Discussion:**

Our results indicate a need for further investigation of the role of *Callithrix* spp. in the transmission cycles of YFV in Brazil. In particular, YFV transmission was observed in a region where viral circulation has not been recorded for decades and thus vaccination has not been previously recommended.

**Conclusions:**

This highlights the need to straighten epizootic surveillance and evaluate the extent of vaccination programmes in Brazil in previously considered “YFV-free” areas of the country.

## Background

Yellow fever (YF) is an infectious disease that results in an acute febrile illness. It is caused by the yellow fever virus (YFV) (genus *Flavivirus*, family *Flaviviridae*) that is transmitted by arthropod vectors. Notable vectors mosquitoes of the genera *Haemagogus* and *Sabethes*, which are involved in the sylvatic cycle in South America, as well as the urban mosquito species *Aedes aegypti* [1, 2]. In Africa, the savannah transmission cycle connects the sylvatic and urban cycles of both humans and monkeys, which are infected by *Aedes* mosquitoes [3].

In Brazil, two distinct disease transmission cycles have been recorded, sylvatic and urban [1]. The sylvatic cycle is endemic to certain regions of South America, including the Amazon region and other areas. Since 1942, with vaccination and vector control programmes of *Aedes aegypti* mosquitoes, the urban cycle was considered eradicated in Brazil [2]. YF outbreaks have been reported every 5–7 years [2]. Prerequisites for epidemic outbreaks include available reservoirs of infection with YFV (such as non-human primates, NHPs) and high vector population densities (such as *Haemagogus* spp. and *Sabethes* spp.) [1].

The primary wild hosts of YFV are non-human primates in both Africa and the Americas. However, African monkeys are more resistant to the virus and hardly die from the infection. In contrast, new world primates can succumb to the virus, which makes them a good epidemiological marker for epidemiological surveillance [4]. In the Americas, species of all genera of NHPs that have been recognized and experimentally infected are sensitive and susceptible to YFV, particularly *Alouatta* spp., *Sapajus* spp. and *Callithrix* spp. [3].

Epizootic outbreaks of YF are considered to be epidemiological markers for cases of YF among humans. The sickness and death of the animals can trigger decisions to expand surveillance and vaccination activities to prevent human outbreaks. The Brazilian Ministry of Health established the NHP Epizootic Events Surveillance System in 1999 to alert of risk YF outbreak, which was incorporated into the Brazilian “Health Unique System” (SUS) in accordance with law 8.080/90 [5]. The surveillance of epizootics in NHPs is useful for delimiting areas of viral transmission, as well as highlighting areas that require greater surveillance, prevention and control [6].

During the 2008–2009 YF outbreak in the State of Rio Grande do Sul (south-eastern Brazil), the surveillance of NHPs was not considered useful for predicting the outbreak and containing the re-emergence of the virus in the region [7]. Like the outbreak in the State of São Paulo, virus detection among NHPs did not serve as an epidemiological marker, but it did facilitate the demarcation of new risk-associated areas [8]. Between January 2016 and December 2017, seven countries and territories in South America reported confirmed YFV cases (Bolivia, Brazil, Colombia, Ecuador, French Guiana, Peru and Suriname). The number of human cases and epizootics collectively reported during this period in these regions was the highest observed in decades [9].

The 2016–2017 YFV epidemic in Brazil accounted for 1659 epizootics, 779 confirmed cases of human YF, and 262 deaths, with most occurring in Southeast Brazil [10]. The epidemic persisted in 2018–2019, but this period was characterized by low transmission in humans [11]. Phylogenetic studies showed that viral isolates of this outbreak were within the same cluster of modern Brazilian strains, which confirmed previous observations. This led to the suspicion that there was no introduction of a new genotype in the country and that only pre-existing strains were transmitted [12, 13]. Indeed, surveillance of epizootic diseases within the surveillance system in previous years has shown the need for monitoring as a tool not only for preventing the spread of the virus within territories but also for improving containment barriers. This previous knowledge led to the intensification of the monitoring response during the 2017 and 2018 outbreaks in Brazil. In order to contribute to the surveillance of YF, the aim of the present study is to describe the laboratory findings of epizootics that occurred in the states of Rio de Janeiro (RJ), Minas Gerais (MG), Espírito Santo (ES), and Bahia (BA) during 2017 and 2018.

## Methods

### Sample collection

NHP specimens were analyzed within the YF surveillance programme at the Flavivirus Laboratory (LABFLA) of the Oswaldo Cruz Foundation (Fiocruz) in Rio de Janeiro. This laboratory is a Brazilian Ministry of Health Regional Reference Laboratory for arboviruses. Diagnostic assays were performed on tissue fragments from NHPs (liver, kidney, brain tissue and whole blood when available) that had been found dead in states in north-eastern (Bahia) and south-eastern Brazil (Rio de Janeiro, Espírito Santo and Minas Gerais). Samples from Minas Gerais were sent only until March 2017. The eligibility criteria for YF detection were that frozen fresh samples were available and accompanied by a notification report from the Ministry of Health (unfortunately, due to the urgency of the surveillance, we could not exclude the notification forms that did not describe the genus of NHPs).

### RNA extraction and rtRT-PCR analysis

Specimens were processed in a biosafety level 3 (BSL3) environment and manipulated under BSL2 conditions after inactivation. Nucleic acid extraction was performed using a MagMAX^TM^ Pathogen RNA/DNA kit (Life Technologies, Carlsbad CA, USA) in accordance with the manufacturer’s instructions. Approximately 30 mg of tissue were disrupted in 600 μl of lysis buffer; an aliquot of 115 μl of the lysate was then mixed with 20 μl of bead mix plus and 65 μl of 100% isopropanol. The extraction was carried out in a KingFisherFlex Automatic Extractor (Thermo Fisher Scientific, Waltham, USA) in accordance with the manufacturer’s instructions.

Negative controls were included in all steps of processing the samples to monitor for possible cross-contamination. To check the RNA isolation efficiency, we used RNase P as an endogenous positive control. The extracted RNA was subjected to TaqMan rtRT-PCR as described by Domingo et al. [14], which targets the highly conserved 5’ noncoding region (5’ NC). The threshold cycle value Cq was employed as an indirect measure to assess the viral load and discriminate positive samples from negatives. Samples with a Cq value ≤ 37 were considered positive.

### Statistical analysis

Baseline characteristics are presented as frequencies of positivity (%) and as medians and means for non-normally distributed continuous data (Cq values). The NHP genus and state of collection were treated as grouping variables. A dataset was composed using an Excel datasheet, reviewed by the researchers to avoid potential misinterpretation, and exported to the R environment. Differences in the median Cq values across the NHP genera were assessed using the nonparametric Kruskal-Wallis analysis of variance. We also used the Mann-Whitney U-test to compare the Cq distribution between the pairs of genera. All statistical tests were two-sided, and *P*-values < 0.05 were interpreted as statistically significant.

## Results

A total of 4198 samples from 2099 NHPs were screened for YFV detection by rtRT-PCR. Between January and December 2017, 1049 suspected NHP cases were analyzed, while 1050 NHPs were analyzed from January to October 2018. The number of the positive samples was 207 (20%) in 2017 and 52 (5%) in 2018. Among them, 77 were NHPs from the state of MG, 156 were from ES, 1177 were from RJ and 689 were from BA.

**Fig. 1:**
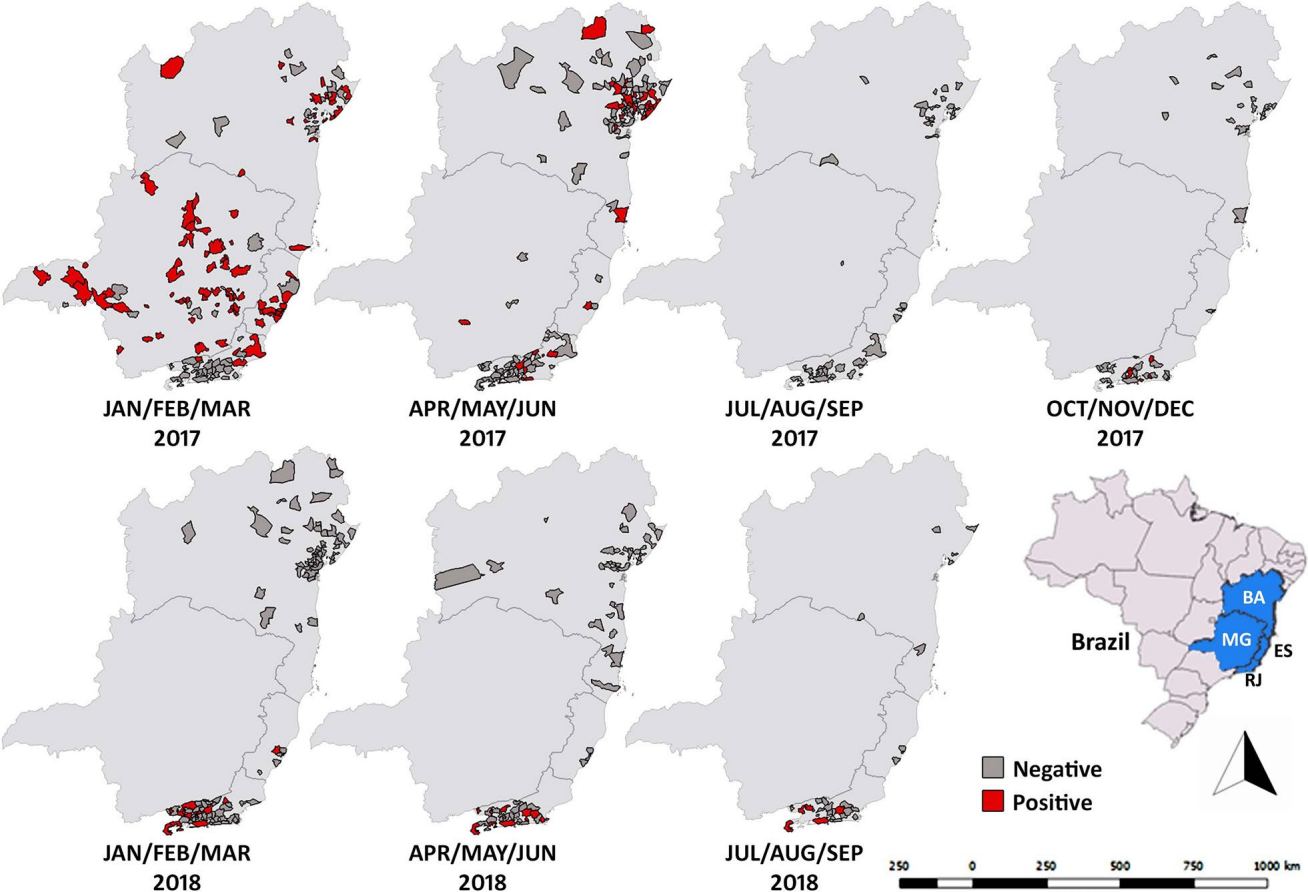
Maps of the states of Espirito Santo (ES), Rio de Janeiro (RJ), Bahia (BA), and Minas Gerais (MG) showing the with distribution of YFV infected and non-infected non-human primates that tested positive and negative for YFV infection. The infections were assessed by rtRT-PCR analysis for each municipality every trimester from January to December 2017 and from January to October 2018. The negatives were suspected cases of NHPs found dead in areas where epizootics were occurring, that tested “not detectable” by RT-PCR. Maps were created using QGIS software version 3.0.0 (Girona; https://qgis.org) and Photoshop

The geographical extent of the YF outbreak in 2017 to 2018 is shown in Fig. 1. Epizootics have been reported in NHPs in these regions. However, in the last decades, virus circulation and NHP epizootics from YFV were not detected in these areas except for MG, which has shown epizootics of NHPs and disease outbreaks since 2000.

**Fig. 2:**
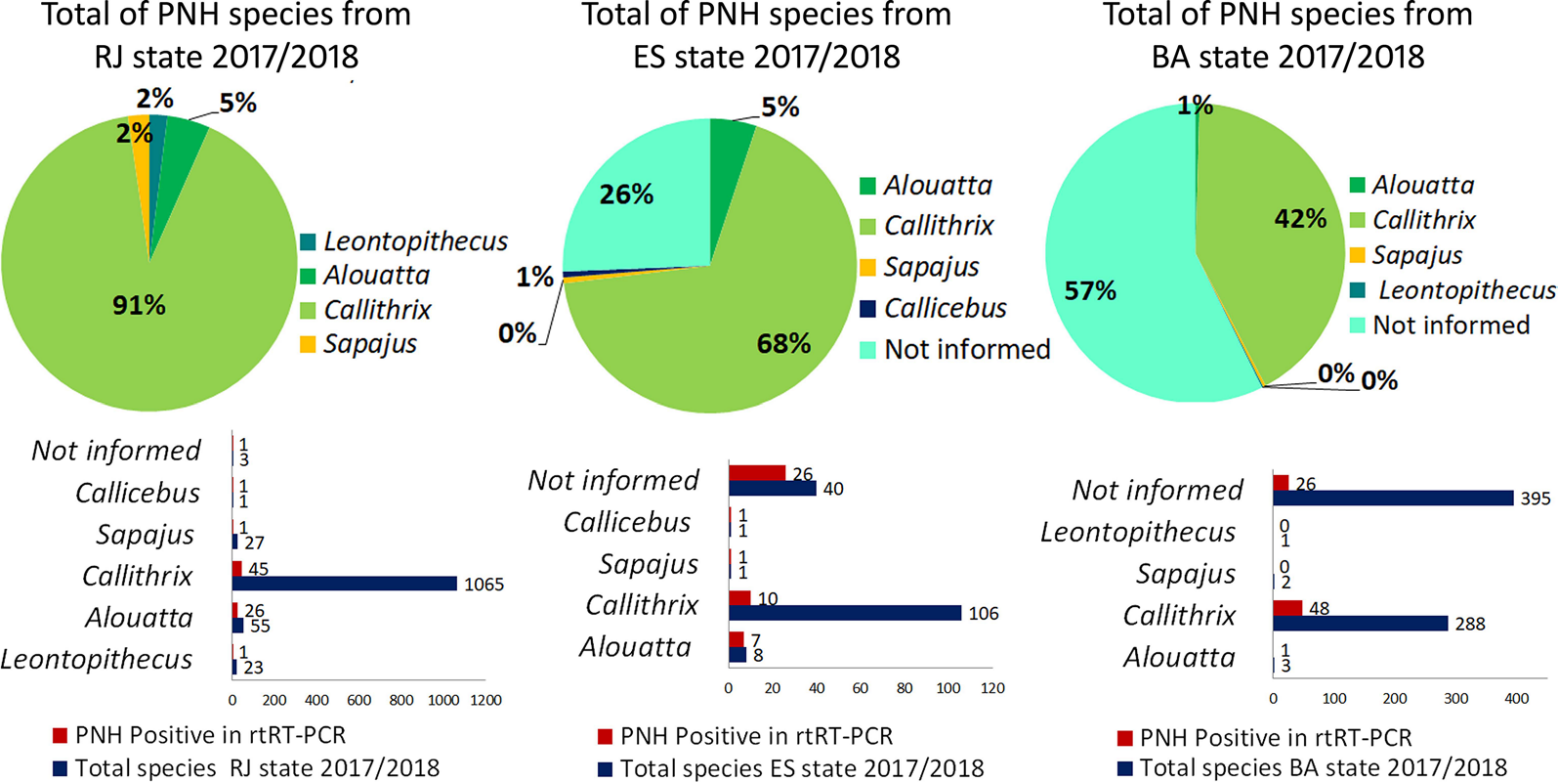
Infected and uninfected animals assessed by rt-RT-PCR analysis according to genus, state, and period. *Abbreviations*: RJ, Rio de Janeiro State; ES, Espírito Santo; BA, Bahia

NHP genus classification data were obtained from epidemiological records that accompanied the samples. Out of the 2099 NHPs analyzed, 1505 (71.7%) were *Callithrix* spp. with 140 (9.3%) positive, 76 (3.6%) were *Alouatta* spp. with 43 (56.6%) positive, 38 (1.8%) were *Sapajus* spp. with 8 (21%) positive, 5 (0.2%) were *Callicebus* spp. with 5 (100%) positive, 24 (1.1%) were *Leontopithecus* spp. with 1 (4.2%) positive, and 448 (21.3%) were from NHPs unidentified to the genus level with 62 (13.8%) positive (Fig. 2). The genus was not determined for many animal samples because the data were not recorded by local surveillance. The samples sent by zoological gardens with identification of the affected species comprised *Aotus* spp., (*n* = 1); *Papio hamadryas* (*n* = 1); and *Papio anubis* (*n* = 1). ES, BA, and RJ had higher frequencies of samples from NHPs of the genus *Callithrix*.

**Fig. 3:**
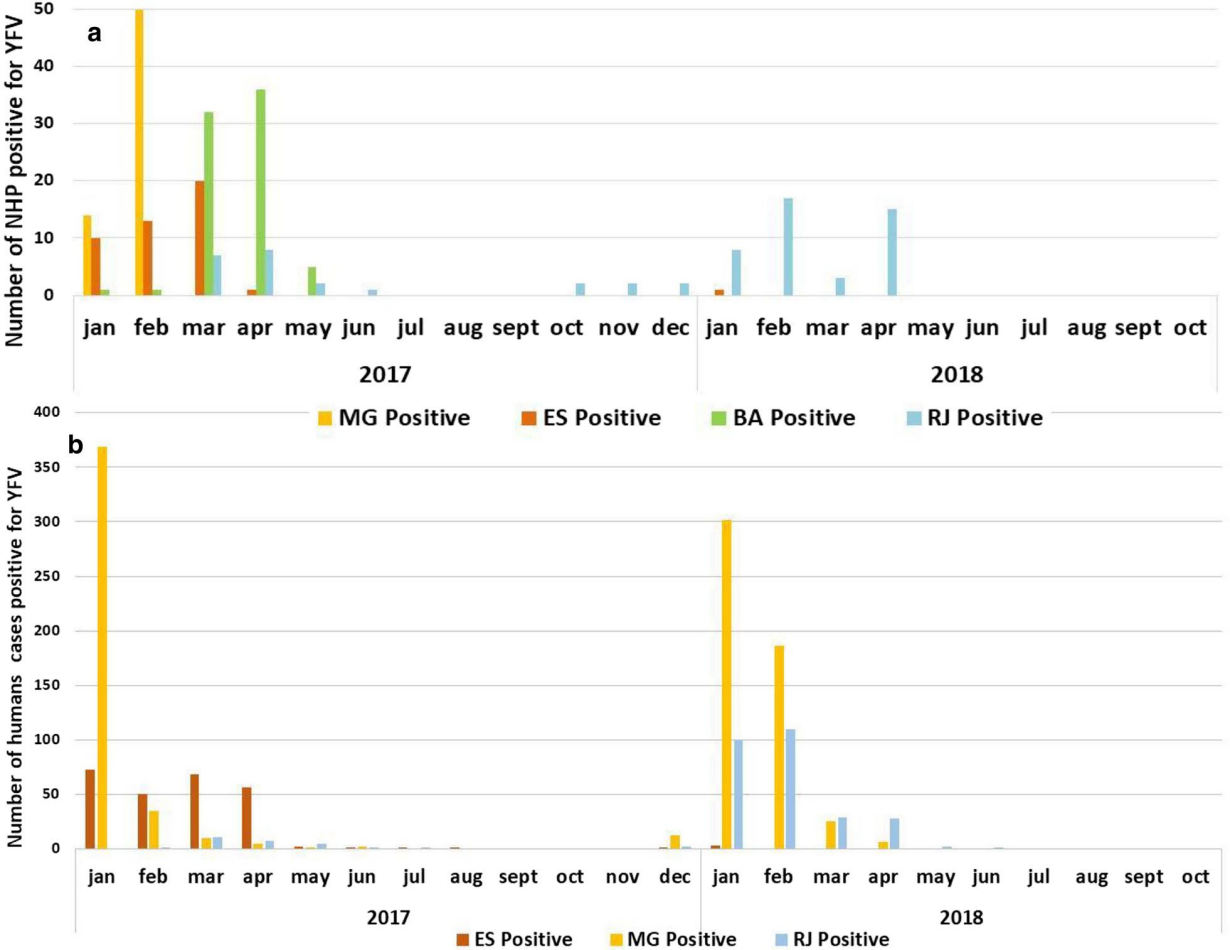
Temporal distribution of confirmed cases of YFV from 2017 to 2018. **a** Number of infected animals assessed by real-time rtRT-PCR analysis in each state and month from January to December 2017 and from January to October 2018. **b** Distribution of human cases confirmed for YFV reported to SVS/MS, by symptom onset and classification from July 2017 to June 2018 in Brazil according to the Brazilian Ministry of Health [10]

The number of NHPs infected with YFV in each state increased primarily from February to March, particularly in MG and ES in 2017 (Fig. 3). In 2018, we observed the same frequency in the months of January to April. However, the highest frequency occurred in February during Carnival (Fig. 3).

**Fig. 4:**
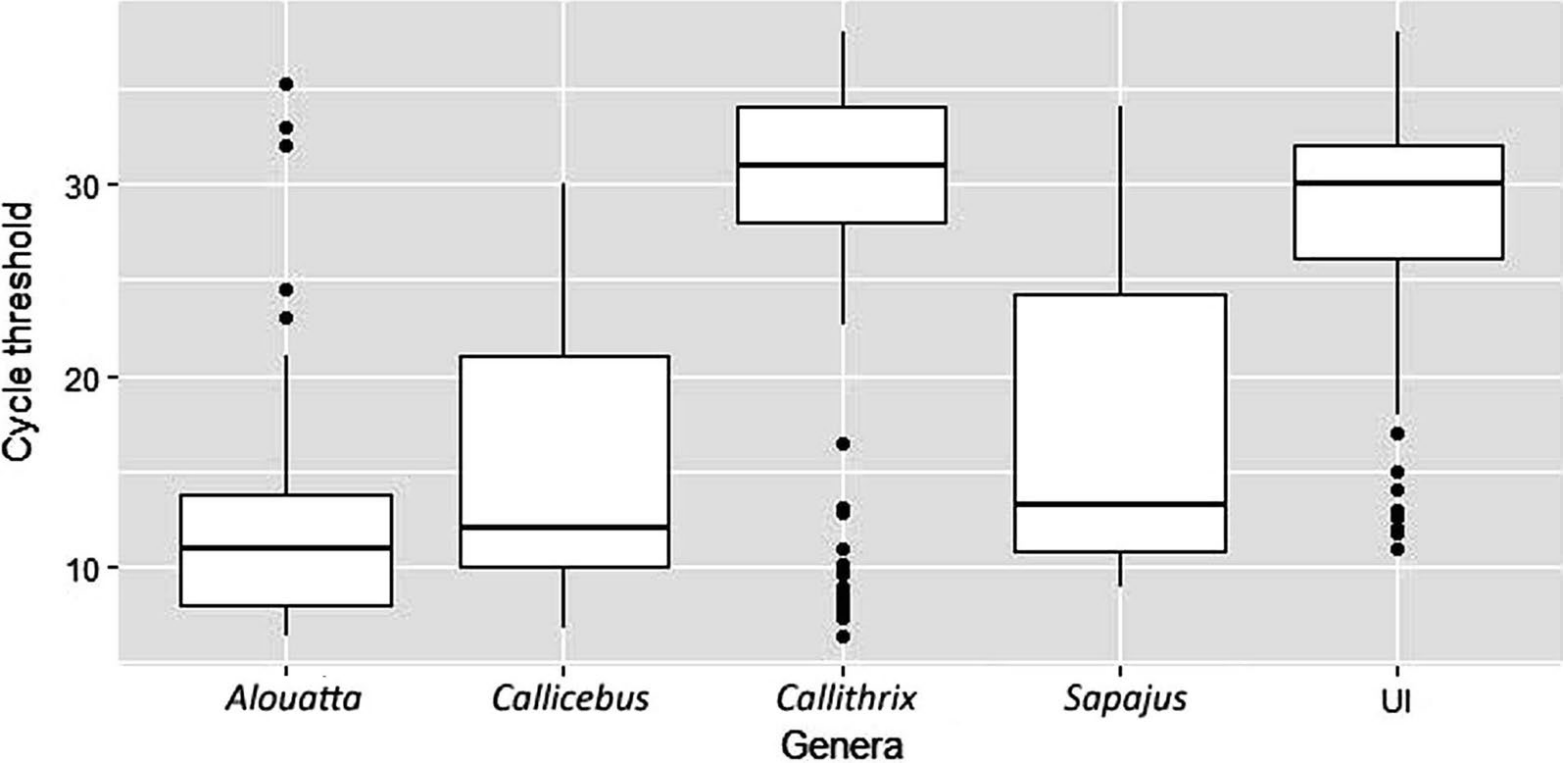
Yellow fever RNA copies measured by quantification cycle threshold (Cq) values according to PNH species. Samples with a Cq ≤ 37 were considered positive. *Abbreviations*: UI, unidentified species

Regarding the molecular detection of YFV using rt-RT-PCR, Cq-values ranged between 6.42–35.26 (mean = 13.04, median = 11.0) in *Alouatta* spp., 6.8–30.0 (mean = 15.96, median = 12.0) in *Callicebus* spp., 6.36–38.0 (mean = 28.16, median = 31.0) in *Callithrix* spp., 9.0–34.0 (mean = 18.15, median = 13.2) in *Sapajus* spp., and 10.9–38.0 (mean = 27.4, median = 30.0) in unidentified species (UI). When comparing Cq-values of the sampled species, the median value was significantly different among the NHP species (Kruskal-Wallis H-test, *χ*^2^ = 65.32, *df* = 4, *P* < 0.0001) (Fig. 4). The analysis showed significant differences in the distribution of Cq-values between *Alouatta* and *Callithrix* (Mann-Whitney U-test, *U* = 843.5, *df* = 2, *P* < 0.0001), *Alouatta* and UI (Mann-Whitney U-test, *U* = 302, *df* = 2, *P* < 0.0001), *Callicebus* and *Callithrix* (Mann-Whitney U-test, *U* = 114.5, *df* = 2, *P* = 0.010), *Callicebus* and UI (Mann-Whitney U-test, *U* = 49, *df* = 2, *P* = 0.011), *Sapajus* and *Callithrix* (Mann-Whitney U-test, *U* = 801.5, *df* = 2, *P* = 0.040) and *Sapajus* and UI (Mann-Whitney U-test, *U* = 133, *df* = 2, *P* = 0.034).

## Discussion

In the present study, howler monkeys (genus *Alouatta*) and marmosets (genus *Callithrix*) were the most frequently infected NHPs. Few cases were encountered in species of the genera *Callicebus* and *Sapajus*. Lion tamarins (genus *Leontopithecus*) were mostly under active surveillance, and only one animal positive for YFV infection was detected in 2018. These results are consistent with previous findings and confirm that species of *Alouatta* and *Callithrix* are more susceptible to the disease than *Sapajus*, *Ateles* and *Saimiri* species in this area [9].

A descriptive study from 2008–2009 [15] reported that although cases of *Callithrix* mortality have been observed, laboratory confirmation was available only for species of the genus *Alouatta*. This may be due to the lower sensitivity of laboratory methods available that time, which were not able to detect low viremia in the specimens of *Callithrix*. An important result when comparing the genera *Alouatta* and *Callithrix* was that significant differences in median viral load were detected. *Callithrix* spp. show a lower viral load, do not develop a fatal YFV infection similar to that reported in humans, and can somehow persist and maintain the possibility of urbanization of YFV. The difference in median values of Cq observed between *Callitrix* and *Allouatta* in this study was also reported in PNHs from the State of São Paulo in south-eastern Brazil [16].

Interaction between marmosets and residents of urban and peri-urban areas is currently restricted to forested areas and urban parks [17]. However, deforestation areas coupled with increased interaction between urban residents and wild species could be a risk factor for the re-emergence of urban YFV epidemics in South America. Anthropophilic mosquitoes *Aedes aegypti* and *Aedes albopictus* are highly susceptible to American and African YFV strains [18]. Therefore, further studies are required to determine whether the genus *Callithrix* imposes a possible relevant risk for the maintenance of sylvatic YF.

The present NHP monitoring results expanded our knowledge of YFV transmission in areas that have not been considered to be at risk and do not have vaccine recommendations. As a result, the Ministry of Health has intensified vaccination campaigns in the affected regions. YF epizootics in Brazil occur every five or seven years, particularly in the Amazon region [2]. However, epizootic events have also been reported outside the Amazon River basin between 2000 and 2010 [19]. The frequency of outbreaks among NHPs is probably a result of the renewal of NHP populations that are susceptible to YFV infection [2]. In 2008–2009, epidemics in Rio Grande do Sul and São Paulo provided evidence of the expansion of YFV transmission to the Southeast, which threatens densely populated areas on the Atlantic coast [20, 21].

Although East and Central Africa regions experienced a resurgence of YF outbreaks [22], there are no reports of epizootics as observed in the Americas. The probable reason is that African monkeys are more resistant to the virus and hardly die from infection but rather become immune [23–25]. Despite reports of YF outbreaks in the Americas, data on NHP species are scarce in countries like Bolivia, Brazil, Colombia, Ecuador, French Guiana, Peru and Suriname [10]. Only a few molecular epidemiological studies in Peru [26] and Trinidad [27] have been reported. This poses a difficulty for the comparison of our findings with those from other Latin American countries.

Our results showed the highest frequency of infected NHPs in MG and ES, followed by BA and RJ. The prevalence of epidemiological-marker NHPs in MG and ES is consistent with human case reports [10, 11]. This reflects the utility and the contribution of a NHPs epizootic surveillance system to provide early warning of disease outbreaks or the prevalence of the disease in affected areas or subgroups of animals. Two studies have described the epidemic scenario in two affected states, ES [28] and Bahia [29]. However, none have assessed the differences prevalence between the most affected NHP genera and the differences in Cqs among them. In 2018, the positive primates were detected in the metropolitan region of RJ to the south, which is different from 2017, where the concentration was higher in the north and center of the metropolitan region. Only one primate has been confirmed in ES.

We have shown that the highest number of positive animals occurred in February to March, starting from the state of MG, followed by transmission to ES and BA, and finally to RJ in 2017. In 2018, we observed the same proportion of positive samples in the months of January to April. However, the highest prevalence occurred during Carnival, when there were several cases and reports of tourists becoming sick or dying, which mainly occurred in the regions of Angra dos Reis and Ilha Grande [30]. These results are different from a previous report in Rio Grande do Sul for the period of 2008–2009, which indicated that the epizootic peak occurred in April 2009 [21]. This behavior may be a response to the seasonality of the disease, which is influenced by factors like weather and vector density, which were different between the epizootics in Rio Grande do Sul and São Paulo in 2017 [19]. One study of YF focused on geo-environmental factors that were studied by spatial and statistical analysis. The results showed that the presence of YFV was associated with rain, altitude, diversity of NHP hosts, and temperature [31]. This emphasizes that environmental aspects may influence different aspects of the transmission of YFV [31].

We observed that approximately 10% of the NHPs were from specimens not identified to the genus level. Of the unidentified animals, more than 60% showed a positive result for YFV. Together with the fact that significant viral load differences were indirectly detected among NHP distinct genera, these observations reinforce the need for genus identification to improve the correlation data between viral load *versus* genus and to clarify the role of the different genera of NHPs in the transmission and maintenance of YF. The difficulty of retrieving more information about animals is a limitation of the present study. The notification reports sent with the samples were missing important information, such as genus, sex and age, shown to be especially relevant in this study. One of the possibilities beyond the intensification of surveillance actions is to establish the best collection of data and information. Protocols for the genomic identification of animals that do not have a completed form in the future can also be established.

Interestingly, out of 288 NHPs from BA, 75 tested positive for YF, while no human cases were confirmed. In total, 42% of these NHPs were from the genus *Callithrix*, and only 1% were *Alouatta* spp.; the remaining 57% had missing genus identification. The role of LABFLA in the Ministry of Health (MoH) network is to investigate epizootics and human cases suspected of yellow fever infection. In this regard, it was not possible to state the relationship between the absence of human cases with the greater presence of monkeys the genus *Callithrix*. However, this is an observation that deserves further investigation considering the low viremia found in species of this genus. In the natural forest habitats of NHPs, mosquitoes transmit arboviruses from infected to naive animals *via* sylvatic transmission cycles (NHP-mosquito-NHP-mosquito, etc.) [24]. Due to the importance of these sylvatic cycles, future investigations are needed into the roles of hosts and vectors in the epidemiology of diseases such as dengue, chikungunya, yellow fever and Zika [24]. Unlike the DENV virus in the sylvatic cycle in Southeast Asia and Africa, which have different ecological and evolutionary lineages [32], YFV in Brazil does not seem to present the same problem according to molecular epidemiological reports carried out in the NHPs of BA [13]. It is always important to consider the possible presence of another pathogen (herpes virus, malaria or *Toxoplasma*) in these animals when infected with the yellow fever virus that can contribute to the death of these animals. Perhaps the low YFV viremia alone cannot cause death of *Callithrix* spp. Considering that the viruses found in different regions and in the NHP and in humans did not show evolutionary differences [13, 33], we reinforce the need for more focused observation of species of this genus, co-infections and their role in the YF outbreaks in Brazil.

## Conclusions

In summary, our approach suggests that the role of the genus *Callithrix* in the sylvatic cycle of YF is an important finding. This genus is present in densely populated urban areas. Thus, the results indicate that special attention is needed to understand and prevent the urban and peri-urban transmission of YFV in Brazil.

## Data Availability

Data supporting the conclusions of this article are included within the article.

## Abbreviations

YFV: yellow fever virus
NHP: non-human primate
rtRT-PCR: real-time reverse transcription polymerase chain reaction
SUS: Health Unique System
RJ: Rio de Janeiro
MG: Minas Gerais
ES: Espírito Santo
BA: Bahia
LABFLA: Flavivirus Laboratory
BSL3: biosafety level 3
BSL2: biosafety level 2
RNA: ribonucleic acid
RNase P: ribonuclease P
5’ NC: 5’ noncoding region
Cq: quantification cycle
CONEP: National Ethical Committee for Research, Ministry of Health, Brazil
CGLAB: General Coordination of Laboratories
SVS/MS: Department of Health Surveillance of the Ministry of Health, Brazil
LASP: Municipals Laboratory of Public Health
CVSLR/Fiocruz: coordination of health surveillance and reference laboratories of the Oswaldo Cruz Foundation
MoH: Ministry of Health, Brazil
